# Cadillac care for the poor... addressing local health emergency around STDs and HIV in an urban community-based medical home

**DOI:** 10.1186/1742-4690-9-S1-P74

**Published:** 2012-05-25

**Authors:** Larisa Kudryashova-Hernandez

**Affiliations:** 1 Ancillary HIV Services at Neighborhood Health Services Corporation, Plainfield, USA

## Background

NHSC, an urban community-based health center in Plainfield, Union County, NJ, USA, provides services to 25,000 uninsured/minority/impoverished patients. Plainfield consistently ranks first and second among the 29 Union County municipalities for Syphilis, Gonorrhea and Chlamydia. Plainfield ranks second for the numbers of HIV/AIDS. There is, therefore, a dire need to address the existing health emergency around STDs and its correlation with HIV.

## Methods

NHSC incorporates a coordinated, proactive, patient-centered approach to integrating STD screening/prevention with primary care in a medical home environment. Risk assessments/screenings are done by clinicians. Those identified suspicious for STD or with STD symptoms/diagnosis receive on-the-spot HIV counseling and Rapid testing. Patients receive immediate treatment intervention for suspected STDs. There are on-going prevention efforts including development of Risk Reduction Plans agreed upon/signed by patients.

## Results

Resulting from the integrated STD/HIV prevention approach: 100% of patients presenting with symptoms/suspicion for STDs received HIV counseling/Rapid testing; 100% received prevention education and free condoms; 100% had Risk Reduction Plans developed and agreed upon; 19 persons were identified STD and HIV positive and were immediately linked to care.

## Conclusions

Integration of STD/HIV prevention with outpatient care under the umbrella of Early Intervention Services allowed to: identify extend of STD/HIV correlation; allow for a seamless one-stop shop prevention-treatment service delivery model; improve patient awareness of on-site prevention/treatment resources.

